# Supplementary material for: Biopsychosocial approach to understanding determinants of depression among men who have sex with men living with HIV: A systematic review
Source: PLoS One. 2022 Mar 14;17(3):e0264636. doi: 10.1371/journal.pone.0264636 (PMC8920233; doi:10.1371/journal.pone.0264636)
Supplement: Attachment — Submitted filename: Response to the reviewers - 14.12.21.docx [file pone.0264636.s002.docx]

Dec 15, 2021

Dear PLOS editors,

Rebuttal Letter to manuscript:

**Biopsychosocial approach to understanding determinants of depression among men who have sex with men living with HIV: A systematic review**

Zul Aizat Mohamad Fisal^1^, Halimatus Sakdiah Minhat^2^, Nor Afiah Mohd Zulkefli^2^, Norliza Ahmad^2^

^1^DrPH Candidate, Faculty of Medicine and Health Sciences, Universiti Putra Malaysia, 43400 Serdang, Selangor, Malaysia

^2^Department of Community Health Sciences, Faculty of Medicine and Health Sciences, Universiti Putra Malaysia, 43400 Serdang, Selangor, Malaysia

Dear Academic Editor Omar Sued and dear reviewers,

We would like to thank you for your comments and recommendations, which gave us the opportunity to improve the paper. In the updated manuscript, we expect to answer all the issues identified. In this document we answer to all the questions highlighted by the reviewers.

Comments are shown in bold font, followed by our answer/comment in normal font. The corrections/changes in the manuscript are displayed through the track changes.

**Editor’s comment**:

**When submitting your revision, we need you to address these additional requirements.**

**1. Please ensure that your manuscript meets PLOS ONE's style requirements, including those for file naming. The PLOS ONE style templates can be found at**

[**https://journals.plos.org/plosone/s/file?id=wjVg/PLOSOne_formatting_sample_main_body.pdf**](https://journals.plos.org/plosone/s/file?id=wjVg/PLOSOne_formatting_sample_main_body.pdf)  **and**

[**https://journals.plos.org/plosone/s/file?id=ba62/PLOSOne_formatting_sample_title_authors_affiliations.pdf**](https://journals.plos.org/plosone/s/file?id=ba62/PLOSOne_formatting_sample_title_authors_affiliations.pdf)

Dear editor, we thank you for the general appreciation of our work and the specific comments given that help to improve our manuscript. The manuscript has followed all the journal requirements.

**2. Thank you for stating the following financial disclosure:**

**“NO”**

**At this time, please address the following queries:**

**a)	Please clarify the sources of funding (financial or material support) for your study. List the grants or organizations that supported your study, including funding received from your institution.**

Dear editor, there is no grant for our work, but we received funding from our institution, which is Universiti Putra Malaysia (UPM).

**b)	State what role the funders took in the study. If the funders had no role in your study, please state: “The funders had no role in study design, data collection and analysis, decision to publish, or preparation of the manuscript.”**

Dear editor, the funders had no role in study design, data collection, and analysis, decision to publish, or manuscript preparation.

**c)	If any authors received a salary from any of your funders, please state which authors and which funders.**

Dear editor, authors received no salary from the funders.

**d)	If you did not receive any funding for this study, please state: “The authors received no specific funding for this work.”**

**Please include your amended statements within your cover letter; we will change the online submission form on your behalf.**

Done. Thank you.

**3. Thank you for stating the following in your Competing Interests section:**

**“NO”**

**Please complete your Competing Interests on the online submission form to state any Competing Interests. If you have no competing interests, please state "The authors have declared that no competing interests exist.", as detailed online in our guide for authors at http://journals.plos.org/plosone/s/submit-now**

**This information should be included in your cover letter; we will change the online submission form on your behalf.**

Done. Thank you.

**4. In your Data Availability statement, you have not specified where the minimal data set underlying the results described in your manuscript can be found. PLOS defines a study's minimal data set as the underlying data used to reach the conclusions drawn in the manuscript and any additional data required to replicate the reported study findings in their entirety. All PLOS journals require that the minimal data set be made fully available. For more information about our data policy, please see http://journals.plos.org/plosone/s/data-availability.**

**Upon re-submitting your revised manuscript, please upload your study’s minimal underlying data set as either Supporting Information files or to a stable, public repository and include the relevant URLs, DOIs, or accession numbers within your revised cover letter. For a list of acceptable repositories, please see http://journals.plos.org/plosone/s/data-availability#loc-recommended-repositories. Any potentially identifying patient information must be fully anonymized.**

**Important: If there are ethical or legal restrictions to sharing your data publicly, please explain these restrictions in detail. Please see our guidelines for more information on what we consider unacceptable restrictions to publicly sharing data: http://journals.plos.org/plosone/s/data-availability#loc-unacceptable-data-access-restrictions. Note that it is not acceptable for the authors to be the sole named individuals responsible for ensuring data access.**

**We will update your Data Availability statement to reflect the information you provide in your cover letter.**

Dear editor, all data are fully available without restriction. Thank you.

**5. Please include your tables as part of your main manuscript and remove the individual files.**

Done. Thank you.

**6. Please review your reference list to ensure that it is complete and correct. If you have cited papers that have been retracted, please include the rationale for doing so in the manuscript text, or remove these references and replace them with relevant current references. Any changes to the reference list should be mentioned in the rebuttal letter that accompanies your revised manuscript. If you need to cite a retracted article, indicate the article’s retracted status in the References list and also include a citation and full reference for the retraction notice.**

Dear editor, the reference list has been corrected and completed.Thank you.

**7. We noticed you have some minor occurrence of overlapping text with the following previous publication(s), which needs to be addressed:**

**-https://journals.lww.com/aidsonline/Fulltext/2019/07150/Mental_health_and_HIV-AIDS__the_need_for_an.1.aspx**

**- https://www.wjpps.com/Wjpps_controller/abstract_id/12341**

**In your revision ensure you cite all your sources (including your own works), and quote or rephrase any duplicated text outside the methods section. Further consideration is dependent on these concerns being addressed.**

Dear editor, all minor occurrence of overlapping text has been addressed. Thank you.

**Reviewer’s comment:**

**1. Is the manuscript technically sound, and do the data support the conclusions?**

**The manuscript must describe a technically sound piece of scientific research with data that supports the conclusions. Experiments must have been conducted rigorously, with appropriate controls, replication, and sample sizes. The conclusions must be drawn appropriately based on the data presented.**

**Reviewer #1: Partly**

Dear reviewer #1, we thank you for the general appreciation of our work, and specific comments given that help to improve our manuscript. The conclusions has been drawn appropriately based on the data presented.

**2. Has the statistical analysis been performed appropriately and rigorously?**

**Reviewer #1: N/A**

**3. Have the authors made all data underlying the findings in their manuscript fully available?**

**The PLOS Data policy requires authors to make all data underlying the findings described in their manuscript fully available without restriction, with rare exception (please refer to the Data Availability Statement in the manuscript PDF file). The data should be provided as part of the manuscript or its supporting information, or deposited to a public repository. For example, in addition to summary statistics, the data points behind means, medians and variance measures should be available. If there are restrictions on publicly sharing data—e.g. participant privacy or use of data from a third party—those must be specified.**

**Reviewer #1: No**

Dear reviewer #1, all data are fully available without restriction. Thank you.

**4. Is the manuscript presented in an intelligible fashion and written in standard English?**

**PLOS ONE does not copyedit accepted manuscripts, so the language in submitted articles must be clear, correct, and unambiguous. Any typographical or grammatical errors should be corrected at revision, so please note any specific errors here.**

**Reviewer #1: No**

Dear reviewer #1, the typographical or grammatical errors has been corrected at revision. The revised manuscript has been proofread by certified proof readers.

**5. Review Comments to the Author**

**Please use the space provided to explain your answers to the questions above. You may also include additional comments for the author, including concerns about dual publication, research ethics, or publication ethics. (Please upload your review as an attachment if it exceeds 20,000 characters)**

**Reviewer #1: I appreciate the opportunity to review this manuscript that approaches a highly relevant topic for public health, such as correlates of depressive symptoms among MSM with HIV. This review is necessary as a systematization of evidence that can guide the development of public policies and interventions, to improve mental health and HIV outcomes in this population. I congratulate the authors for choosing this topic and for a remarkable work. However, I consider that there is still room for improvement of this manuscript so that it is suitable for publication.**

Dear reviewer #1. Thank you. We try to improve and do our best.

**General overview**

**-Several issues were found regarding writing, grammar and spelling throughout the whole manuscript (e.g., typos, syntactic and grammar errors, missing words, etc.). A thorough proofread is required.**

**-It is noteworthy that citation style changes throughout the manuscript. Adequacy to the journal requirements and guidelines should be thoroughly revised.**

Dear reviewer #1. The revised manuscript has been proofread by certified proof readers. The citation erros has been corrected based on Vancouver style.

**Abstract**

**-In the first sentence, I recommend using the present tense “are” rather than the past tense “were”.**

Dear reviewer #1, we have changed “were” to “are”.

**-The first sentence in the Conclusions paragraph would be better located in the Results paragraph.**

Dear reviewer #1, the sentence has been locted in result paragraph.

**Introduction**

**-The use of the term “gay” is recommended instead of the term “homosexual”. In paragraph 5 the term “HIV-seropositive MSM” is used. I recommend using “MSM living with HIV” which is actually the term used throughout the manuscript, for consistency.**

Dear reviewer #1, the term “MSM living with HIV” has been used throughout the manuscript, for consistency.

**-Paragraph 3, sentence 2: It should be “MSM are vulnerable…”**

Dear reviewer #1, we have followed you suggestion here.

**-The introduction may benefit from a clearer order and organization of information. At some parts, it is somewhat repetitive and there is some disconnection between paragraphs. A possible reorganization is, for example, the following: current paragraph 1 is fine as an introduction of the main variable, second paragraph should be a summary of determinants of depression both in the general population and among MSM and people with HIV and presentation of the BPS approach (which is currently distributed between paragraphs 3 and 4, both paragraphs could be merged and integrated), third paragraph should be about consequences of depression among MSM with HIV (currently paragraph 5), the fourth paragraph could be a conclusion about the importance to address depression among MSM living with HIV (currently paragraph 2), the sixth paragraph is fine with conclusion and objectives.**

Dear reviewer #1, we have followed you suggestion here. The second paragraph is the summary of determinants of depression both in the general population and among MSM and people with HIV and presentation of the BPS approach (Paragraphs 3 and 4, has been merged and integrated and become the second paragraph). The third paragraph are the consequences of depression among MSM with HIV. The fourth paragraph is the conclusion about the importance to address depression among MSM living with HIV (taken from paragraph 2).

**-In the Introduction, the BPS construct is introduced as BPS approach, which I find it is a better term than construct. The authors may consider using the term BPS approach also in the rest of the manuscript.**

Dear reviewer #1, the term BPS approach has been used in the rest of the manuscript including in the title.

**Methods**

**-Quality assessment: The remaining articles are the selected articles?**

Dear reviewer #1, yes, the remaining articles are the selected articles. The sentence has been corrected.

**Results:**

**-Study selection: reasons for exclusion, as listed in this section, should be expressed in a clearer way so that readers can accurately understand why a set of articles was excluded from analysis. For example, one reason is “the general PLHIV population”. That would not be the reason exactly, but “inclusion of general PLHIV population or not MSM population in the study sample”.**

Dear reviewer #1, the reasons for exclusion has been changed to: the inclusion of HIV-negative participants in the study sample, and not MSM population in the study sample.

**-Study characteristics: “Studies’ characteristics” would be a more appropriate title for this section as it describes the characteristics of the studies included in this review, and not the characteristics of the review itself.**

Dear reviewer #1, The title “Studies’ characteristics” has been used.

**-As previously mentioned, I recommend using the term “MSM living with HIV” or “MSM with HIV”, instead of HIV positive or seropositive MSM (or simply HIV MSM, as in the Discussion, please avoid using this term). I recommend consistency in the use of terms.**

Dear reviewer #1, the term “MSM living with HIV” has been used throughout the manuscript, for consistency.

**-I also recommend using the term “social” instead of “sociological”, as it is not related to sociology but to society.**

Dear reviewer #1, the term “social” instead of “sociological” has been used throughout the manuscript.

**-Within the biological factors, it is stated that “ART initiation improved depression”. This means that depression was reduced, it decreased, it was associated with a reduction of depressive symptoms? Perhaps the sentence could be expressed in a clearer way. The same for viral load, it is not clear if it is associated with increments or reduction of depressive symptoms. I suggest expressing the relations between factors and depression in a clearer way, indicating if they are associated with increased or reduced odds of depression. This same recommendation applies for psychological and social factors.**

Dear reviewer #1,

With regards to biological, psychological, and social factors, the relations between factors and depression has been described in a clearer way, indicating if they are associated with increased or reduced odds of depression/ increased or decreased depression/depressive symptoms.

**-Within the psychological factors, it is stated that stigma is associated with increased odds of depression. However, it is not mentioned what kind of stigma: HIV-related stigma, stigma related to sexual orientation (being gay or bisexual) or other kind? I recommend clarifying this.**

Dear reviewer #1,

The type of stigma has been described as enacted HIV-related stigma.

**-Regarding the CES-D, please revise the correct name of the instrument for greater accuracy.**

**-Some of the instruments listed measure “presence of depressive symptoms” and not “depression” (which is a specific disorder and diagnosis). That is, they screen for depressive symptoms but do not allow to arrive to a diagnosis of depression. This is the case, for example, of the CES-D. In that sense, it can be questioned that the present study is actually systematizing and reviewing factors associated with presence of depressive symptoms, and not factors associated with depression (which is not the same).**

Dear reviewer #1, the brief justification of accepting tools for masurement of depression has been described. For example with regards of CES-D: Following the test objectives, the CES-D provides cut-off scores; for example, a score of 16 or higher can aid in identifying persons who are at risk for clinical depression, with good sensitivity and specificity, as well as a high level of internal consistency. My references as below.

<https://www.apa.org/pi/about/publications/caregivers/practice-settings/assessment/tools/depression-scale> & <https://journals.plos.org/plosone/article?id=10.1371/journal.pone.0155431>).

However, I understood that it is debatable and I will humbly accept your expert decision.

**Discussion**

**-Among the biological factors, older age was a protective factor. Given the vulnerability that younger people exhibit and that it is also a key population regarding HIV and mental health, this is a result that is worth mentioning and explaining in the discussion section.**

Dear reviewer #1.Thank you. The age factor has been discussed.

**-In the “enacted stigma” subsection, it is not clear what “same-sex sexual identity” means, I understand that the authors meant simply “sexual identity”. In this section, it should be clarified what kind of stigma studies refer to.**

Dear reviewer #1, the term “sexual identity was used” and stigma refers to enacted HIV-related stigma.

**-In the “current smoker” section, it is stated that “The number of life-years lost is…, respectively”. However, it is not clear “respectively” to what or who is referring.**

Dear reviewer #1, im sorry for the mistake. The correct sentences is “Smoking was linked to more than 60% of fatalities in PLHIV, whereby they lose more life-years due to smoking, with 12.3 years lost to smoking than 5.1 years lost to HIV”.

**-There are other social factors that are worth mentioning and discussing which have not been included in the Discussion section, such as unemployment and migration. In general, I recommend expanding the Discussion with the inclusion of other relevant factors that have been identified as determinants of depressive symptoms (e.g., unemployment, migration, age, self-efficacy, social support). Although evidence may be weak or moderate for some of them, they are relevant to be considered and analyzed for theoretical or practical reasons.**

Dear reviewer #1, unemployment, born overseas, age, self-efficacy, social support has been added in discussion.

**-Limitations: I would not say “incomparability”, but “limited comparability”, as studies were actually compared.**

Dear reviewer #1, “limited comparability” has been chosen.

**-The fact that studies from different countries were included not only implies demographic differences between patients. It also implies different cultural and legal contexts, with deep variations in the acceptance of gay and bisexual (GB) identities and rights. This influences the levels of social stigma (one of the main factors identified in this study). Some of these countries have laws protecting GB people’s rights whereas others do not. This should be acknowledged.**

Dear reviewer #1, the concern has been acknowledge briefly and concisely.

**-“Foreign language” refers to non-English language? Foreign language depends on the native language of the authors and readers. I agree that this choice introduced bias as most studies come from English speaking countries and there is little evidence from other regions. I would explicitly mention this.**

Dear reviewer #1, yes I mean non-English language.

**-Conclusion: Regarding the first sentence, the determinants that were identified were more than those that are mentioned. I would mention that these were the determinants with the strongest evidence.**

Dear reviewer #1, this sentence has been added: “The determinants of depression with the strongest evidence among MSM living with HIV were…”

**-Recommendations: I suggest mentioning more explicitly, actions to fight stigma in general (e.g., enactment of laws to protect GB people’s rights) and in healthcare services (e.g., services that acknowledge, affirm, and validate diverse sexual identities). Elimination of stigma is mentioned too broadly in the recommendations perhaps, and it deserves a more detailed and expanded mention as one of the key determinants of depression among MSM.**

Dear reviewer #1, your suggestion has been added in recommendation.

**-Table 1, study 4: the date is incomplete.**

Dear reviewer #1, the date has been inserted.

**-Table 1, study 1: the aim is to study predictors of anxiety and generalized stress. What was the role of depression in this study and why was it included if depression was not the outcome?**

Dear reviewer #1, Im sorry for the mistake. the objectives was “to identify and compare risk and protective factors for depression, anxiety, and generalized stress” (Heywood & Lyon, 2016).

**-In table 2, I recommend placing the studies in the same order as Table 1 to facilitate integration of the information from both tables by the reader, as both tables are actually related.**

Dear reviewer #1. The table has been edited for greater sysyemization. The studies in table 2 has been placed in the same order as table 1.

**-According to Table 2, many factors or determinants of depression that were identified in the present review were drawn from the study of Irving et al. (2018). For that reason, one would argue that the integration of results across studies was limited, and that this manuscript is, in some sense, a summary or reproduction of results of other few studies. Relevant factors that are mentioned in other studies (e.g., internalized stigma, which is mentioned in 3 studies) are barely taken into consideration and analyzed in this review (despite being a very important, repeatedly mentioned factor). A greater systematization of findings is required so that this manuscript is not a mere reproduction of results of a few studies, because that adds little value and significant contribution to the field.**

Dear reviewer #1. The relevant factors that are mentioned in other studies (e.g., age, unemployment, internalized stigma, born overseas, self-efficacy, social support) has been added.

**6. PLOS authors have the option to publish the peer review history of their article (what does this mean?). If published, this will include your full peer review and any attached files.**

Dear reviewer #1. Yes, I agree, if published, this will include my full peer review and any attached files.

**If you choose “no”, your identity will remain anonymous but your review may still be made public.**

**Do you want your identity to be public for this peer review? For information about this choice, including consent withdrawal, please see our Privacy Policy.**

Dear reviewer #1. Yes, I want our identity to be public for this peer review.

Your consideration of accepting this manuscript for publication and cooperation on this matter is greatly appreciated.

Thank you.

Yours sincerely,

Zul Aizat Mohamad Fisal, MD

Faculty of Medicine and Health Sciences,

Universiti Putra Malaysia
